# Proteomic 2D-DIGE Analysis of Milk Whey from Dairy Cows with *Staphylococcus aureus* Mastitis Reveals Overexpression of Host Defense Proteins

**DOI:** 10.3390/microorganisms8121883

**Published:** 2020-11-28

**Authors:** Shaimaa Abdelmegid, David Kelton, Jeff Caswell, Gordon Kirby

**Affiliations:** 1 Department of Biomedical Sciences, Ontario Veterinary College, University of Guelph, Guelph, ON N1G 2W1, Canada; sabdelme@uoguelph.ca; 2 Department of Population Medicine, Ontario Veterinary College, University of Guelph, Guelph, ON N1G 2W1, Canada; dkelton@ovc.uoguelph.ca; 3 Department of Pathobiology, Ontario Veterinary College, University of Guelph, Guelph, ON N1G 2W1, Canada; jcaswell@uoguelph.ca

**Keywords:** mastitis, bovine, proteomics, whey, milk proteins, *Staphylococcus aureus*, 2D-DIGE

## Abstract

Bovine mastitis remains a primary focus of dairy cattle disease research due to its considerable negative economic impact on the dairy industry. Subclinical mastitis (SCM), commonly caused by *Staphylococcus aureus*, lacks overt clinical signs and the diagnosis is based on bacteriological culture and somatic cell counts of milk, both of which have limitations. The main objective of this study was to identify, characterize and quantify the differential abundance of milk whey proteins from cows with *S. aureus* SCM compared to whey from healthy cows. Using two-dimensional differential gel electrophoresis (2D-DIGE) coupled with liquid chromatography and tandem mass spectrometry, 28 high-abundant proteins were detected in whey from mastitic milk, 9 of which had host defense functions. These included acute phase proteins involved in innate immunity and antimicrobial functions (e.g., serotransferrin, complement C3, fibrinogen gamma-B chain and cathepsin B), and proteins associated with the immune response to pathogens (e.g., polymeric immunoglobulin receptor-like protein, MHC class I antigen and beta-2-microglobulin). These results provide a unique 2D map of the modulated milk proteome during *S. aureus* mastitis. The broader importance is that the identified proteins, particularly those with host-defense biological functions, represent potential candidate biomarkers of subclinical mastitis in dairy cows.

## 1. Introduction

Bovine mastitis is one of the most prevalent and costly diseases currently affecting dairy herds. The negative economic impact is primarily due to lowered milk production, penalties for high somatic cell count (SCC), discarded milk, increased culling rate or death from infection, and treatment costs. More than 80% of costs associated with mastitis are due to lowered milk production and discarded milk [1,2].

*Staphylococcus aureus* is one of the most frequently isolated contagious mastitis pathogens causing either clinical or subclinical mammary gland infection. Subclinical infection is problematic in dairy cattle because it goes undetected clinically which leads to damage to the mammary tissues and subsequent decrease in milk production [3]. Moreover, subclinical mastitis often leads to persistent and chronic infections because the bacteria have the ability to survive within the mammary epithelial cells [4,5]. The inflammatory response during establishment of the infection is characterized by a number of changes in milk composition due to infiltration of cellular components such as neutrophils, macrophages and soluble immune factors such as complement proteins and acute phase proteins which together synchronize to clear the infection and the pathogen [6,7]. Because these innate immune mediators are usually pathogen-dependent and play an important role in early stages of infection their identification can offer insights into the host inflammatory and immune responses during mastitis.

Currently, there are no effective and efficient detection tests available to identify cattle with *S. aureus*-induced subclinical mastitis. Recently, proteomics has been widely applied to elucidate pathogenic mechanisms of mastitis during natural and experimental infections in different animal species [8,9,10]. While specific cytokine levels and the effects of the innate immune response during bacterial mastitis have been documented [7,8], changes in milk protein expression profiles during naturally occurring subclinical *S. aureus* intramammary infection requires further investigation in order to identify valid biomarkers with the potential to faciliate diagnosis.

The aim of this study was to investigate the changes of the protein profiles in milk whey in response to naturally occurring subclinical intramammary infection with *S. aureus* with the goal of identifying putative biomarkers for diagnostic purposes. In particular, the present study describes the differential abundance of whey proteins as proteomic signatures of *S. aureus*-positive whey fractions compared to uninfected milk samples. To our knowledge, this is the first study that utilizes two-dimensional differential gel electrophoresis (2D-DIGE), to characterize protein abundance in milk whey from cows with *S. aureus* mastitis. The results also provide insight into the role of milk proteins in host–pathogen interaction during *S. aureus* intramammary infection.

## 2. Materials and Methods

### 2.1. Cows and Sample Collection

Milk samples were obtained from 12 lactating cows from the dairy herd at the Livestock Research and Innovation Centre—Dairy Facility of the University of Guelph in Elora, Ontario, Canada. We have previously performed a high-throughput, in-depth proteomic analysis on these samples using a gel-free approach with liquid chromatography–tandem mass spectrometry (LC–MS/MS) and label-free protein quantification [9]. Cows were in mid- to late-lactation and ranged in age from 4 to 8 years. Six cows were culture-positive for *S. aureus* with a high somatic cell count of more than 2 × 10^5^ cells/mL which had a previous history of subclinical mastitis i.e., no systemic or localized signs of mastitis and no history of major systemic diseases. The control group included six healthy cows that were microbiologically negative for *S. aureus* with low SCC of less than 2 × 10^5^ cells/mL with no previous history of clinical mastitis three months preceding sample collection. The cows were also selected based on the results of clinical examination (absence of systemic and local signs) to exclude clinical mastitis. A quarter of the milk samples (30 mL) were collected aseptically, transported on ice and immediately mixed with a protease inhibitor cocktail (Roche Diagnostics GmbH, Mannheim, Germany) in preparation for proteomic analysis. Microbiological examination and SCC were performed within 24 h. The use of all animals in this study was approved by the Animal Care and Use Committee of the University of Guelph.

### 2.2. Somatic Cell Counts (SCC) and Microbiological Examination

Somatic cell counts were determined using a commercial automated cell counter (DeLaval Cell Counter DCC; DeLaval Canada, Sussex, NB, Canada). The bacteriological analysis was performed according to the standard procedures of the National Mastitis Council [10]. Briefly, a loopful of each quarter sterile milk sample was plated on Columbia blood agar (CBA) supplemented with 5% sheep red blood cells, incubated at 37 °C and examined 24 h and 48 h for positive or negative results based on bacterial colony growth. *S. aureus* bacteria were identified by culture characteristic on selective media, Gram-staining and biochemical reactions [11].

### 2.3. Sample Processing and Two-Dimensional Differential Gel Electrophoresis (2D-DIGE)

For the preparation of whey, milk samples were centrifuged at 3000× *g* and 4 °C for 30 min, and the fat layer was removed with a spatula. The skimmed milk was decanted into a clean tube, centrifuged again at 45,000× *g* and 4 °C for 30 min and the supernatant collected and stored at −80 °C. The protein concentration of the whey fractions was estimated using a BCA Protein Assay (BCA Protein Assay kit, Pierce™, ThermoFisher Scientific, Ottawa, ON, Canada) and 50 µg of whey protein was labeled with 400 pmol cyanine dyes, Cy3 (control group) and Cy5 (mastitic group) (Amersham CyDye DIGE Fluors, GE Healthcare, Montreal, QC, Canada). Equal amounts of all experimental samples were properly combined to create the internal pooled standard, which was labeled with the Cy2 cyanine dye. The labeled protein samples were then mixed and diluted in rehydration buffer (8 M urea, 2 M thiourea, 2% CHAPS, 65 mM dithiothreitol, 0.8% ampholyte) and applied to 24-cm IPG strips (pH 3 to 10, non-linear; GE Healthcare, Montreal, QC, Canada) by passive rehydration overnight at room temperature. After rehydration, IPG strips were focused on an Ettan IPGphor IEF system at 20 °C (GE Healthcare, Montreal, QC, Canada) using a gradient voltage increase for a total of about 90,000 Vh for 20 h. After focusing, IPG strips were reduced in equilibration buffer I (6 M urea, 2% SDS, 1.5 M Tris-HCl pH 8.8, 30% glycerol, and 2% (*w*/*v*) dithiothreitol), at room temperature for 20 min, then alkylated in equilibration buffer II (6 M urea, 2% SDS, 1.5 M Tris-HCl pH 8.8, 30% glycerol, and 2.5% (*w*/*v*) iodoacetamide) at room temperature for 20 min. The IPG strips were run in the second dimension on 10% to 20% precast Tris-HCl polyacrylamide gels (Jule Biotechnologies, Inc. Milford, CT, USA) in 0.1% *w/v* SDS, 25 mM Tris–HCl pH 8.3 as running buffer for 200 V for 17 h.

Gels were scanned on a Typhoon 9410 scanner (GE Healthcare, Montreal, QC, Canada). All images were analyzed with DeCyder software 6.5 (GE Healthcare, Montreal, QC, Canada) and differential in-gel analysis (DIA) modules (GE Healthcare, Montreal, QC, Canada) for the detection and matching of spots, while statistical analysis of protein level changes was performed with the DeCyder BVA (biological variation analysis) module. The resultant images of control and mastitic whey proteomes were compared by calculation of fold changes in differential abundance and statistically evaluated with the DeCyder BVA module. A false discovery rate (FDR) of 1% was applied to minimize the number of false-positive results.

Protein spots were characterized as differentially abundant between control and mastitic groups if showing a difference in fold change of ≥2-fold, with a statistically significant variation (*p* ≤ 0.05). Using preparative 2-D gels, the differentially abundant proteins were separated and matched with the analytical gels. For optimal matching, 50 µg of proteins were labeled with Cy5 as described above and then mixed with 550 µg of non-labeled proteins on 24-cm IPG strips with a pH non-linear gradient of 3–10 (GE Healthcare, Montreal, QC, Canada). All strips were rehydrated, focused, and subjected to second dimension electrophoresis as described above. After electrophoresis, all gels were fixed in 10% methanol-7% acetic acid for 40–60 min and post-stained overnight in SYPRO Ruby stain (Lonza Bioscience, Walkersville, MD, USA). Gels were destained in 10% methanol–7% acetic acid for 40 min at room temperature and then imaged on a Typhoon scanner. Matching between 2D DIGE and 2D gels was performed using Decyder analysis software and differential in-gel analysis (DIA) modules and a spot-picking list was generated and exported to an Ettan Spot Picker (GE Healthcare, Montreal, QC, Canada). The gel plugs were excised and placed into wells of 96-well plates.

### 2.4. In-Gel Digestion and Liquid Chromatography–Tandem Mass Spectrometry (LC–MS/MS) Analysis

Gel plugs excised from the 2D gels were subjected to tryptic digestion. Briefly, gel plugs were destained at room temperature for 1 h in 25 mM ammonium bicarbonate–50% acetonitrile. Gel plugs were dehydrated in 100% acetonitrile for 5 min at room temperature and then dried to completeness in a vacuum centrifuge for 20 min at 22 °C. Plugs were rehydrated in 15 μg/mL of trypsin (Sigma-Aldrich, St. Louis, MO, USA) in 25 mM ammonium bicarbonate and allowed to digest for 16 h at 37 °C. After digestion, peptides were extracted with 50% acetonitrile-5% trifluoroacetic acid at room temperature for 1 h. Peptides were concentrated in a vacuum centrifuge for 20 min at 22 °C. Samples were then analyzed on a linear ion trap-Orbitrap hybrid analyzer (LTQ Orbitrap, ThermoFisher Scientific, Ottawa, ON, Canada) outfitted with a nanospray source and EASY-nLC split-free nano-LC system (ThermoFisher Scientific, Ottawa, ON, Canada). Lyophilized peptide mixtures were dissolved in 0.1% formic acid and loaded onto a 75 μm × 15 cm PepMax RSLC EASY-Spray column filled with 2 μM C18 beads (ThermoFisher Scientific, Ottawa, ON, Canada) at a pressure of 800 BAR. Peptides were eluted over 60 min at a rate of 250 nl/min using a 0 to 35% acetonitrile gradient in 0.1% formic acid. Peptides were introduced by nano electrospray into an LTQ-Orbitrap hybrid mass spectrometer (ThermoFisher Scientific, Ottawa, ON, Canada). The instrument method consisted of one full MS scan (400–1400 *m*/*z*) in the Orbitrap mass analyzer with an automatic gain control target of 500,000 with a maximum ion injection of 100 ms followed by one microscan, and a resolution of 100,000. Ten data-dependent MS/MS scans were performed in the linear ion trap using the ten most intense ions at 35% normalized collision energy. The MS and MS/MS scans were obtained in a parallel fashion. In MS/MS mode automatic gain control targets were 10,000 with a maximum ion injection time of 50 ms. Minimum ion intensity of 10,000 was required to trigger an MS/MS spectrum. The dynamic exclusion was applied using a maximum exclusion list of 500 with one repeat count with a repeat duration of 30 s and exclusion duration of 20 s.

### 2.5. Protein Identification

The following database searching parameters were applied. All MS/MS samples were analyzed using Sequest (ThermoFisher Scientific, Ottawa, ON, Canada; version 1.4.0.288) and X! Tandem (The GPM, thegpm.org; version CYCLONE) (2010.12.01.1). Sequest was set up to search Uniprot_Bos_Taurus_Feb232015.fasta assuming the digestion enzyme trypsin. X! Tandem was set up to search the Uniprot_Bos_Taurus_Feb232015 database also assuming trypsin. Sequest and X! Tandem were searched with a fragment ion mass tolerance of 0.60 Da and a parent ion tolerance of 10.0 PPM. Deamidation of asparagine and glutamine, oxidation of methionine and carbamidomethylation of cysteine were specified in Sequest as variable modifications. Scaffold (version Scaffold_4.4.1.1, Proteome Software Inc., Portland, OR, USA) was used to validate MS/MS-based peptide and protein identifications. Peptide identifications were accepted if they could be established at greater than 95.0% probability. Peptide probabilities from Sequest were assigned by the Scaffold Local FDR algorithm. Peptide probabilities from X! Tandem were assigned by the Peptide Prophet algorithm with Scaffold delta-mass correction. Protein identifications were accepted if they could be established at greater than 99.0% probability and contained at least 2 identified peptides. Protein probabilities were assigned by the Protein Prophet algorithm. Proteins that contained similar peptides, and could not be differentiated based on MS/MS analysis alone, were grouped to satisfy the principles of parsimony. Proteins sharing significant peptide evidence were grouped into clusters.

### 2.6. Gene Ontology (GO) and Pathway Enrichment Analyses

To investigate the biological and clinical value of the identified differentially abundant proteins in the mastitic group through 2D-DIGE and mass spectrometry methods, in silico functional analyses were performed. Enrichment analyses for Gene Ontology (GO) terms were performed. The proteins were categorized based on their biological processes (BP), cellular components (CC) and molecular function (MF) using the Database for Annotation, Visualization and Integrated Discovery (DAVID) v6.8 software (http://david.abcc.ncifcrf.gov/) [12]. Pathway enrichment analysis was performed using the Kyoto Encyclopedia of Genes and Genomes (KEGG) [13] to investigate the interaction between the differentially abundant proteins.

## 3. Results

Proteomic analysis of whey fractions from normal and *S. aureus* mastitic bovine milk samples was performed using combined 2D-DIGE and MS procedures. Milk sample grouping was undertaken based on specific microbiological culture and somatic cell counting. Typical morphological and biochemical properties of *S. aureus* were identified including large, creamy hemolytic colonies on CBA, positive coagulase production within 18 h and sugar–alcohol fermentation.

In order to identify protein signatures of each experimental group, the analysis was undertaken on 6 biological replicates in each group for a total of 6 gels. Only protein spots accurately detected and matched in all gels run on samples collected from all the biological replicates were considered for further identification. Analysis of the 2-D DIGE images with DeCyder software (GE Healthcare) through the Biological Variation Analysis (BVA) module allowed gel-to-gel matching and statistical analysis of protein abundance changes between samples (control and mastitic) across the 6 different gels by comparison with the pooled internal standard.

**Figure 1:**
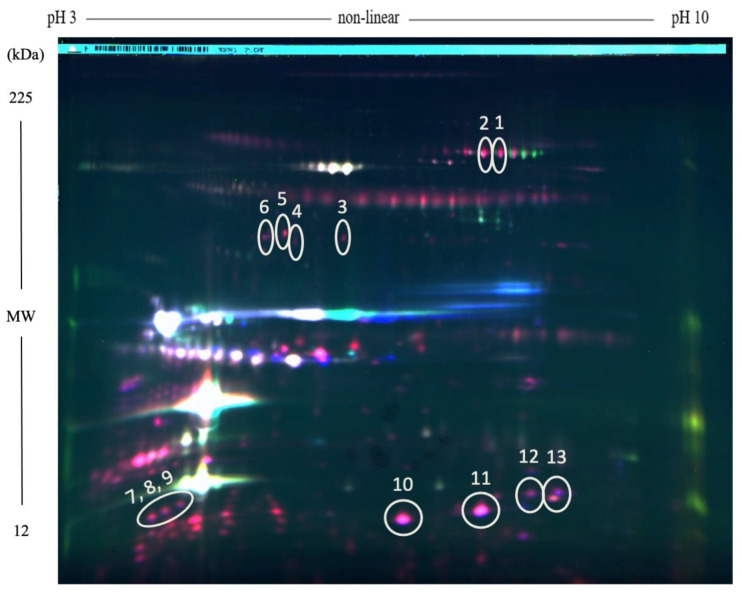
Proteomic analysis of milk whey proteins from healthy cows and cows with with *S. aureus* subclinical mastitis. Two-dimensional differential gel electrophoresis (2D-DIGE) composite image shows differentially-represented proteins in mastitic milk (red spots) compared to milk from healthy controls (green spots). Proteins with similar levels in both groups are represented by white spots. Gel spot numbers correspond to those indicated in Table 1.

Results of statistical analysis indicated significant differential abundance for numerous protein spots in the *S. aureus*-positive group compared to the control group as shown in the representative overlay 2-D DIGE image (Figure 1). In total, 22 differentially abundant spots that were significantly altered two-fold or greater (*p* < 0.05) were selected and picked from the preparatory gel and then further identified using LC–MS/MS analysis. Twenty-eight proteins were identified, 9 of which have a host defense-associated biological function. All the statistically significant spots were highly abundant in the mastitic whey samples and no low-abundant proteins were detected. The identified spots and their corresponding proteins that were modulated or high-abundant in association with *S. aureus* infection are summarized in Some spots were visible on the analytical 2D-DIGE gel images; however, they were undetected in the preparative gel possibly due to the relative lower protein concentration in the preparative gel, which may have resulted in difficulty in spot picking. Multiple spots were often identified representing isoforms of the same protein which might reflect possible post-translational modifications.

In addition to the identification of host proteins in mastitic whey, we also detected proteins of bacterial origin when the results of LC–MS/MS analysis were screened against a *S. aureus* reference proteome database [14] (Table 2). The identified proteins were detected in four spots on the 2D-DIGE gels from mastitic whey. These spots are labelled A, B, C and D and they are not indicated in Figure 1. Because the fold change and total spectral counts of these proteins were less than the bovine proteins, no further analysis was performed to quantify the identified bacterial proteins.

To further investigate the biological and clinical value of the differentially abundant proteins identified in the mastitic group, GO functional annotations and enrichment analysis were performed and the proteins were categorized based on their biological processes (BP), cellular components (CC) and molecular function (MF) using DAVID analysis Differentially abundant proteins that were significantly enriched at a *p*-value <0.05 for each category are presented in Table 3.

There were 30 GO terms identified for the BP category of which the top five were maintenance of location, cellular component organization, cellular component assembly, response to stimulus and response to stress. There were only five GO terms for the CC category which were extracellular region, extracellular space, macromolecular complex and protein complex. There were also only five GO terms for the MF category which included protein binding, oxygen binding, lipid binding and cytoskeletal protein binding.

Pathway enrichment analysis performed using the KEGG pathway to investigate the interaction between the differentially abundant proteins indicated the following: *S. aureus* infection pathway (bta05150) was enriched with complement C3 and fibrinogen gamma-B chain (FGG); Antigen processing and presentation pathway (bta04612) was enriched with Cathepsin B (CTSB) and MHC class I antigen (BOLA-NC1); Complement and coagulation cascades pathway (bta04610) was enriched with FGG and C3 and ECM–receptor interaction pathway (bta04512) was enriched with COL1A1 and DAG1.

## 4. Discussion

This study highlights the differential abundance of the proteomic signatures of *S. aureus*-positive whey fractions compared to the non-infected fractions and provides an insight into understanding the role of milk proteins in host-pathogen interaction during *S. aureus* intramammary infection. During intramammary infection, damage to the milk-secreting tissues occurs which results in a reduction of the synthetic capacity of the mammary epithelial tissues that becomes manifested clinically by decreased milk production. In the early stages of infection, the damage is minimal and reversible. The outcome of bacterial invasion of the mammary gland, whether it is clearance or establishment and persistence of the infection, is mainly dependent upon the effectiveness of the host defense response that occurs immediately after initial infection.

Bovine milk contains a high concentration of proteins with wide-ranging functions. Although dominated by the six major milk proteins (α_S1_-casein, α_S2_-casein, β-casein, κ-casein, β-lactoglobulin, and α-lactalbumin), accumulated evidence has shown that milk also contains a range of low-abundance proteins which are associated with host defense functions that form a significant first line of defense against the invading pathogens. The detection and profiling of the low-abundance proteins is challenging due to the biological complexity and high dynamic range of the constituents of bovine milk. However, advances in proteomic technologies, improvement of bovine protein sequence databases and increased efforts to identify protein biomarkers for early detection of intramammary infections have been able to overcome many of these challenges. This is reflected in several studies investigating the host defense response to different environmental and contagious pathogens such as *E. coli*, *Streptococcus uberis* and *S. aureus* that cause clinical or subclinical mastitis in different animal species [8,9,15,16,17,18,19]. While many studies have investigated the host defense response at the transcriptomic level of mammary epithelial cells during intramammary infection, few have focused on proteomic changes in milk in dairy cows with *S. aureus* mastitis [9,15]. To the best of our knowledge, this is the first study profiling the modulation of the bovine milk whey proteome during natural infection with *S. aureus* utilizing 2D-DIGE coupled with LC–MS/MS analysis.

The 2D-DIGE technique is a robust quantitative method that allows direct comparison of spots between mastitic and healthy samples on the same gel, thus eliminating inter-gel variability. The DIGE workflow also provides a platform for relative quantitation of intact proteins that are differentially labeled with fluorescent dyes that improves the sensitivity of detection and statistical reliability of proteins that are differentially abundant.

Identification of serotransferrin and serum albumin in mastitic whey suggests that changes in vascular permeability results in a compromised barrier between milk and blood leading to exudation of serum proteins into the milk [20,21]. Previous proteomic studies reported that serotransferrin and serum albumin were elevated in response to other bacterial infections resulting in mastitis [8,22,23,24].

Some of the proteins which were differentially abundant in mastitic whey such as fibrinogen gamma-B chain and complement (C3) indicate the involvement of the complement and coagulation cascades. A recent study involving iTRAQ-proteomic analysis of mammary tissue from cows naturally infected with *S. aureus* suggested that clinical and subclinical mastitis might cause disturbances in coagulation due to fibrin accumulation and subsequent clot formation [17]. Previously, complement C3 was identified in normal whey only [25], however, in the present study it was identified in both mastitic and normal whey but with higher abundance in mastitic samples. This is in agreement with other studies that reported the essential biological role of the complement cascade in innate immunity by eliciting chemoattraction and activation of leukocytes, opsonization and killing of pathogens [8,22,26,27].

The identification of polymeric immunoglobulin receptor-like protein (PIgR) with higher abundance in mastitic whey is particularly intriguing. PIgR is a transmembrane glycoprotein which is expressed in many mucosal epithelium cell types including mammary epithelial cells (MECs) [28,29]. Recently, several studies showed that PIgR is one of the pattern recognition receptors (PRRs) which are expressed and activated through the MECs to respond to bacterial infection and so-called pathogen-associated molecular patterns (PAMP). PIgR plays an active role in transporting IgG into the lactating mammary glands and facilitates the translocation of IgA across the MECs. Once at the apical side of MECs, PIgR is cleaved to release IgA into the alveolar lumen which suggests that this receptor may contribute to bridging the innate and the adaptive immune response in MECs [28,30,31].

Beta-2-microglobulin is a component of MHC class I molecules and is associated with transport of serum IgG into the mammary gland of cattle and is responsible for the transfer of IgG into colostrum [32]. Proteomic 2DE analysis of whey samples from cows and sheep with mastitis including subclinical mastitis have revealed elevated levels of beta-2-microglobulin [18,21,33,34].

The abundance of cathepsin B in mastitic whey is consistent with previous studies which reported its increase in milk quarters infused with lipoteichoic acid (LTA) from *S. aureus,* in whey from cows with subclinical mastitis naturally infected with S. aureus or infected with *Streptococcus uberis* [9,20,35]. Intracellular granules of polymorphonuclear neutrophils (PMNs) contain several neutral and acidic proteases such as elastase and cathepsins B and G that have the ability to kill different pathogens that cause mastitis [30]. Additionally, these proteases contribute to the elevated SCC and the increased proteolysis of caseins during infection [35,36].

The roles of apolipoproteins (APO) in lipid transport and as a component of high-density lipoproteins (HDL) have been well characterized [37]. Recently, several investigations revealed other roles, such as anti-inflammatory functions, for apolipoproteins particularly APO A-I (APOA1). This includes inhibition of neutrophil activation and suppression of inflammatory cytokine release following *E.coli* intramammary infection suggesting a role in immune response modulation during mastitis [38,39]. The immune-modulatory functions of APOA1 were also reported during *S. uberis* and *S. aureus* mastitis but to a lesser extent [18,26]. In the current study, we observed that apolipoprotein-E (APO-E) was highly abundant in mastitic milk. Others have detected a high-abundance of APO-E in the cerebral spinal fluid of cows showing signs of BSE [40]. However, the specific role of APO-E in inflammation, particularly during mastitis, requires further investigation.

The relatively high abundance of different forms of casein (αS1-casein, αS2-casein, β-casein, κ-casein) in the whey of mastitic animals was unexpected in this study in view of our efforts to partially deplete caseins to enrich low abundant whey proteins. As reported previously [8,41], the concentration of caseins decreases during mastitis while the whey proteins (β-lactoglobulin, and α-lactalbumin) may increase or decrease if the epithelial cells lose their synthetic functions due to tissue damage. The increase of caseins in the current analysis may reflect proteolysis due to activation of plasmin during infection rather than a higher level of production of caseins [20,39].

Several protein spots were characterized as bovine serum albumin (BSA) in the mastitic whey in the current study. The multiple isoforms of the BSA which were detected on 2D gels have the same molecular weight (69 kD), however, they have different pI values which is similar to results demonstrated in a previous study [42]. In contrast, others have reported shifts in both molecular weight and pI of BSA isoforms in mastitic whey due to *E. coli* infection [22]. A possible explanation for the abundance of multiple forms of BSA is proteolysis as a result of trypsin digestion during sample preparation for MS analysis.

The results of bioinformatics analysis reveal differential expression of proteins that reflect the biological processes and cellular activities occurring in the mammary gland during *S. aureus* infection, specifically the host defense biological functions. The categories generated by GO functional annotations and enrichment analysis signify responses of mammary tissues to the stresses initiated by the presence of the bacteria notably maintaining and regulating cell form and function, responding to stressful external stimuli and wounding, regulating cell death, and facilitating wound healing by promoting angiogenesis. While similar GO categories were identified in our previous assessment of these samples by shotgun proteomic analysis [9], more specific GO categories were identified in that analysis reflecting the host response including antimicrobial responses, the acute phase response, cytokine production and pathogen recognition functions.

When bacteria gain access to mammary tissues, a typical first line of host defense is activation of the innate immune response either by recognition of pathogen molecules or by upregulation of the local resident immune components in order to combat an invading pathogen. In the present study, several host defense proteins including PIgR, complement C3, fibrinogen gamma-B chain, APO-E and cathepsin B were differentially expressed. To the best of our knowledge, this is the first time that high abundances of complement C3, PIgR and APO-E have been identified in mastitic milk. While differential expression of the antimicrobial peptide Cathepsin B was also detected in our previous shotgun proteomic analysis of these samples [9], other host defense proteins were identified including those with roles in antimicrobial activity (i.e., cathelicidin-4 peptidoglycan recognition protein, lactoperoxidase and lactotransferrin), the acute phase response (i.e., haptoglobin) and pathogen-recognition (i.e., lipopolysaccharide-binding protein, monocyte differentiation antigen and chitinase-3-like protein 1). Changes in the expression levels of innate immune mediators, acute phase proteins and antimicrobial peptides could represent biomarkers of the host defense response that predict early intramammary infection with specific pathogens. It is likely that a panel of biomarkers will be necessary to enable the detection of subclinical stages of *S. aureus* mastitis with high sensitivity and specificity.

In conclusion, increased knowledge of the host immune response to different mastitis pathogens is an essential prerequisite for the detection and diagnosis of subclinical mastitis. The results of the current study present, for the first time, 2D-DIGE analysis that elucidates the proteomic changes that occur in bovine milk naturally infected with *S. aureus*. There were inherent limitations in the gel-based approach used in this study that resulted in the low number of proteins identified in our proteomic analysis compared to our previous study in which LC–MS/MS and label-free proteomic analyses were used [9]. Possible reasons include insufficient separation of low-abundance proteins and acidic and basic proteins from high-abundance, high molecular weight proteins likely due to the high complexity and the high dynamic range of proteins present in bovine milk. Moreover, the limited sensitivity of SYPRO Ruby staining of preparatory gels made it difficult to pick all differentially abundant proteins identified on analytical 2D-DIGE gels. Nonetheless, the majority of high-abundance proteins such as complement C3, serotransferrin, polymeric immunoglobulin receptor, cathepsin B and fibrinogen gamma-B chain have relevant biological functions related to host defense and acute phase responses. These data provide information about biomarkers that could be validated and subsequently used in future studies for the detection of intramammary infection at subclinical stages.

## Figures and Tables

**Table 1:** List of proteins identified by 2D-DIGE/liquid chromatography–tandem mass spectrometry (LC–MS/MS) analysis that are highly abundant in milk from cows with subclinical *Staphylococcus aureus* mastitis.

Function	Protein Name	Accession Number	Gel Spot No.	*p*-Value	Fold Change Relative to Controls	Mr (kDa)	Unique Peptides	Sequence Coverage (%)
**Host-Defense Proteins**	Serotransferrin	Q29443|TRFE_BOVIN	1	0.007	3.3	77.6	3	50
	Polymeric immunoglobulin receptor	P81265|PIGR_BOVIN	1	0.007	3.3	82.4	12	16
			2	0.003	5.3	82.4	13	15
	Fibrinogen gamma-B chain	F1MGU7|F1MGU7_BOVIN	3	0.012	3.5	50.2	22	56
	Complement C3	G3X7A5|G3X7A5_BOVIN	4	0.0001	7.5	187.1	29	17.5
			5	0.025	6.2	187.1	43	23.7
	Beta-1,4-galactosyltransferase 1	P08037|B4GT1_BOVIN	4	0.0001	7.5	44.8	5	13.7
	MHC class I antigen	H6V5G4|H6V5G4_BOVIN	4	0.0001	7.5	38.8	6	26.3
	Apolipoprotein E	A7YWR0|A7YWR0_BOVIN	4	0.0001	7.5	35.9	4	17.7
	Cathepsin B	P07688|CATB_BOVIN	4	0.0001	7.5	36.6	13	34.4
	Beta-2-microglobulin	P01888|B2MG_BOVIN	13	0.001	20.3	13.6	5	25.4
**Major Milk Proteins**	Serum albumin	P02769|ALBU_BOVIN	3	0.012	3.5	69.2	5	8.5
			4	0.0001	7.5	69.2	27	51.6
			5	0.025	6.2	69.2	15	29.8
			6	0.002	11.3	69.2	11	20.8
			11	0.003	13.7	69.2	7	13.7
	Alpha-S1-casein	P02662|CASA1_BOVIN	7	0.005	17.1	24.5	4	32.7
			8	0.002	24.7	24.5	7	35
			9	0.002	13.7	24.5	21	48.1
	Alpha-S2-casein	P02663|CASA2_BOVIN	6	0.002	11.3	26.0	3	16.2
	Beta-casein	P02666|CASB_BOVIN	11	0.003	13.7	25.1	5	21.4
			12	0.009	40.4	25.1	7	25
			13	0.001	20.3	25.1	6	21.9
	Kappa-casein	P02668|CASK_BOVIN	4	0.0001	7.5	21.2	5	30
			6	0.002	11.3	21.2	4	25.8
**Structural and Metabolic proteins**	Collagen alpha-1 (I) chain	P02453|CO1A1_BOVIN	3	0.012	3.5	138.9	4	3.1
	Actin, cytoplasmic 1	P60712|ACTB_BOVIN	4	0.0001	7.5	41.7	23	60
			5	0.025	6.2	41.7	11	33.3
			6	0.002	11.3	41.7	29	72.8
			7	0.005	17.1	41.7	3	8.5
	Actin, cytoplasmic 1	P60712|ACTB_BOVIN	10	0.002	21.9	41.7	13	41.6
			11	0.003	13.7	41.7	6	24.3
			13	0.001	20.3	41.7	6	17.9
	Dystroglycan	F1N7D7|F1N7D7_BOVIN	4	0.0001	7.5	97.3	5	7.26
	N-acetylglucosamine-1-phosphotransferase subunit gamma	Q58CS8|GNPTG_BOVIN	4	0.0001	7.5	33.7	5	19.3
	Hemoglobin subunit alpha	P01966|HBA_BOVIN	10	0.002	21.9	15.1	4	31.7
	Thymosin beta-4	P62326|TYB4_BOVIN	10	0.002	21.9	5	3	54.5
	Histone H2B	F1MUD2|F1MUD2_BOVIN	10	0.002	21.9	13.9	3	38.1
	Histone H1.2	P02253|H12_BOVIN	10	0.002	21.9	21.3	3	11.7
**Unknown**	Putative uncharacterized protein	A5D7Q2|A5D7Q2_BOVIN	4	0.0001	7.5	51.6	4	9.65

**Table 2:** List of proteins identified by searching a *S. aureus* reference proteome database.

Spot Number	Protein Name	Protein Accession Numbers	Unique Peptide Count	Sequence Coverage
**A**	50S ribosomal protein L19	RL19_STAA1	2	17.20%
**A**	Orotate phosphoribosyltransferase	PYRE_STAAC,PYRE_STAAS	2	10.80%
**A**	Immunoglobulin G-binding protein A	SPA_STAA8	8	23.10%
**B**	ATP-dependent Clp protease ATP-binding subunit ClpL	CLPL_STAAR	5	16.00%
**B**	DNA topoisomerase 4 subunit B	PARE_STAEQ	5	13.30%
**B**	Hyaluronate lyase	HYSA_STAA8	7	13.40%
**B**	Putative hemin import ATP-binding protein HrtA	HRTA_STAAR	5	24.40%
**B**	Septation ring formation regulator EzrA	EZRA_STAEQ	5	12.60%
**B**	Protein draper	DRPR_DROME	6	16.10%
**C**	DNA-directed RNA polymerase subunit beta (Fragment)	RPOC_STAAU	5	12.10%
**C**	60 kDa chaperonin	CH60_STAAU	5	20.40%
**D**	Immunoglobulin G-binding protein A	SPA_STAA8	10	26.60%
**D**	Staphopain B	SSPB_STAAS	2	10.90%
**D**	Transcription termination/antitermination protein NusG	NUSG_STAA8	2	14.30%
**D**	Lactose phosphotransferase system repressor	LACR_STAA8, LACR_STAAR	2	18.30%
**D**	Acetylglutamate kinase	ARGB_STAAR	2	22.70%

Database name: Uniprot_Staphylococcus-aureus_Feb192017.fasta. Peptide thresholds: 95.0% minimum, Protein thresholds: 99.9% minimum and 2 minimum unique peptides.

**Table 3:** Gene Ontology (GO) terms over-represented in significantly abundant whey proteins due to *S. aureus* mastitis.

Categories	Biological Processes	Count ^1^	*p*-Value ^2^
**Cellular form**	GO:0044085~cellular component biogenesis	5	0.009
	GO:0065003~macromolecular complex assembly	4	0.017
	GO:0016043~cellular component organization	8	0.002
	GO:0022607~cellular component assembly	5	0.005
	GO:0009653~anatomical structure morphogenesis	5	0.008
	GO:0043933~macromolecular complex subunit organization	4	0.02
	GO:0051270~regulation of cell motion	3	0.008
	GO:0048856~anatomical structure development	6	0.021
	GO:0048646~anatomical structure formation involved in morphogenesis	3	0.033
	GO:0051651~maintenance of location in cell	2	0.028
	GO:0051235~maintenance of location	3	0.001
**Regulation of cellular process**	GO:0032879~regulation of localization	4	0.008
	GO:0051179~localization	9	0.011
	GO:0048523~negative regulation of cellular process	5	0.021
	GO:0048519~negative regulation of biological process	5	0.03
	GO:0065008~regulation of biological quality	5	0.029
	GO:0051049~regulation of transport	3	0.037
	GO:0001649~osteoblast differentiation	2	0.038
**Cell Death**	GO:0042981~regulation of apoptosis	4	0.017
	GO:0043065~positive regulation of apoptosis	3	0.022
	GO:0043068~positive regulation of programmed cell death	3	0.022
	GO:0010942~positive regulation of cell death	3	0.022
	GO:0043067~regulation of programmed cell death	4	0.018
	GO:0010941~regulation of cell death	4	0.018
**Response to stimulus**	GO:0009605~response to external stimulus	4	0.019
	GO:0050896~response to stimulus	8	0.005
	GO:0006950~response to stress	6	0.006
	GO:0009611~response to wounding	3	0.043
**Angiogenesis**	GO:0001944~vasculature development	3	0.022
	GO:0001568~blood vessel development	3	0.021

Count ^1^ = the number of proteins enriched in the GO term using DAVID (Database for Annotation, Visualization and Integrated Discovery) software. *p*-value ^2^ = Absolute *p*-value where significantly (*p* ≤ 0.05) annotated proteins were considered.
